# Chronic Low Back Pain in Nurses: Effects On Life and Work

**DOI:** 10.1192/j.eurpsy.2026.11680

**Published:** 2026-07-31

**Authors:** N. Kotti, A. Kchaou, I. Ben Hnia, N. Rmadi, M. Hajjaji, K. J. Hammami

**Affiliations:** Occupational Health, Hedi Chaker University Hospital, Sfax, Tunisia

## Abstract

**Introduction:**

Chronic low back pain represents a considerable public health concern due to its substantial social, economic, and occupational impact. It is one of the most common reasons for medical consultation, particularly among healthcare professionals. This condition negatively influences not only caregivers’ quality of life (QoL) but also the quality of care delivered to patients.

**Objectives:**

The purpose of this study was to estimate the prevalence of chronic low back pain and to explore its effects on nurses’ quality of life.

**Methods:**

A descriptive, cross-sectional observational study was carried out using an anonymous self-administered questionnaire among nurses attending the Occupational Medicine Department at Hedi Chaker University Hospital in Sfax during a three-month period. Quality of life in nurses with chronic low back pain was evaluated through the Oswestry Disability Index.

**Results:**

A total of 120 nurses were invited to participate, and 106 completed the questionnaire appropriately, yielding a response rate of 88.33%. Women represented 64.2% of respondents. The mean age was 40 ± 9.8 years, with professional experience ranging from 1 to 29 years (mean: 16.43 ± 10 years). During the past 12 months, 94.3% reported experiencing low back pain, and 29% indicated persistent symptoms lasting more than three months. Sick leave related to low back pain was reported by 48% of participants. Functional limitations were also frequent: 27.6% could not lift heavy objects from the floor, 51.7% experienced difficulty sitting, and 48.2% reported reduced tolerance to prolonged standing. Mobility restrictions due to pain were noted in 58.5% of cases. More than half of the participants (55.2%) presented with minimal disability.

**Conclusions:**

The prevalence of chronic low-back pain remains high among healthcare workers. Appropriate prevention programs are therefore essential. Future studies are needed to further evaluate the psychological and professional consequences of this pathology.

**Disclosure of Interest:**

None Declared

